# Participatory methods used in the evaluation of medical devices: a comparison of focus groups, interviews, and a survey

**DOI:** 10.1186/s12913-024-10887-3

**Published:** 2024-04-12

**Authors:** Kas Woudstra, Marcia Tummers, Catharina J. M. Klijn, Lotte Sondag, Floris Schreuder, Rob Reuzel, Maroeska Rovers

**Affiliations:** 1 Department of Health Evidence, Radboudumc, Nijmegen, Netherlands; 2 Department of Operating Rooms, Radboudumc, Nijmegen, Netherlands; 3 Department of Neurology, Donders Institute for Brain, Cognition, and Behavior, Radboudumc, Nijmegen, Netherlands

**Keywords:** Comparison, Qualitative methods, Stakeholder involvement, Medical devices, Neurology, Surgery

## Abstract

**Background:**

Stakeholder engagement in evaluation of medical devices is crucial for aligning devices with stakeholders’ views, needs, and values. Methods for these engagements have however not been compared to analyse their relative merits for medical device evaluation. Therefore, we systematically compared these three methods in terms of themes, interaction, and time-investment.

**Methods:**

We compared focus groups, interviews, and an online survey in a case-study on minimally invasive endoscopy-guided surgery for patients with intracerebral haemorrhage. The focus groups and interviews featured two rounds, one explorative focussing on individual perspectives, and one interactive focussing on the exchange of perspectives between participants. The comparison between methods was made in terms of number and content of themes, how participants interact, and hours invested by all researchers.

**Results:**

The focus groups generated 34 themes, the interviews 58, and the survey 42. Various improvements for the assessment of the surgical procedure were only discussed in the interviews. In focus groups, participants were inclined to emphasise agreement and support, whereas the interviews consisted of questions and answers. The total time investment for researchers of focus groups was 95 h, of interviews 315 h, and survey 81 h.

**Conclusions:**

Within the context of medical device evaluation, interviews appeared to be the most appropriate method for understanding stakeholder views since they provide a scope and depth of information that is not generated by other methods. Focus groups were useful to rapidly bring views together. Surveys enabled a quick exploration. Researchers should account for these methodological differences and select the method that is suitable for their research aim.

**Supplementary Information:**

The online version contains supplementary material available at 10.1186/s12913-024-10887-3.

## Background

Medical devices form an intricate part of the healthcare system. Novel medical devices like robots, nano-technologies, and e-health platforms carry the promise of improving healthcare systems [1]. As medical devices become more pervasive and complex, it is essential to develop and apply these technologies so that they solve the most pressing medical problems in global healthcare systems. Multiple guidelines and regulations exist that stimulate a practice of medical device research and development that is aimed at solving critical health problems [2–4]. In these documents, one of the recommendations is to actively involve a diverse selection of stakeholders in the research and development process of medical devices. This should lead to better informed decisions during evaluation: aligning devices with the views, needs, and values of stakeholders like medical professionals or patients. This, in turn, can optimise the use of resources spent on research, development, implementation, or use [5].

There are several methods for stakeholder involvement but these have not been compared against the background of their suitability for medical device evaluation purposes. Studying methods within this context is important, because there are some typical requirements in medical device evaluation. Methods should yield relevant information for research or development choices, preferably foster agreement among stakeholders regarding the future development and implementation of the medical device, and be feasible in terms of resources. By relevant information we mean: any information that helps to understand what features a device should have or how research into a device should be conducted to meet the needs and values of stakeholders. This could involve effectiveness, functionalities, ease of use, affordability, or possible spill overs. By analysing stakeholder needs and practices and making consequent design changes, medical devices can become more valuable [6, 7]. Fostering agreement is important to ensure that a device is sufficiently endorsed to make implementation successful. This requires interaction between stakeholders to find common ground [8]. In entrepreneurial settings where resources are limited and the life-cycle of medical devices is relatively short, development trajectories generally cannot be too long and costly [9]. Participatory methods can—on the other hand – give insights into development, evaluation, and implementation issues that can occur, and therefore possibly save costs. Due to these unique conditions, it is important to specifically analyse participatory methods in the context of medical device evaluation or development. Some general comparisons of interviews, focus groups, and surveys exist terms of effect on outcomes exist, but these comparisons are not directly applicable to medical devices, nor are they compared all three together [10–12].

Interviews and focus groups are the most often-used methods in participatory research of medical devices and therefore we compare these in this study [13]. Surveys are chosen because they are also used in qualitative research and because they methodologically differ on various aspects from interviews and focus groups [13]. Qualitative surveys offer open text boxes and therefore researchers cannot ask follow-up questions, and there is no direct interaction between researchers and participants. Therefore, we aimed to investigate how focus groups, interviews, and a survey compare in terms of the number of relevant themes they provide, interaction between stakeholders, and time-investment, when conducted in the context of the evaluation of a medical device.

## Methods

### Comparison in one clinical case

We simultaneously employed and compared three participatory methods: focus groups, interviews, and a survey, in an empirical case-study on minimally-invasive endoscopy-guided surgery for patients with intracerebral haemorrhage (ICH). ICH is the deadliest stroke subtype, with a 30-day case fatality of 40%, and of the patients surviving many live with severe disability [14]. To improve outcome of patients with ICH, an innovative endoscopic device has been developed that has the potential of removing the haemorrhage by minimally-invasive surgery. A systematic review and meta-analysis of randomised trials investigating surgical treatment of ICH showed that minimally-invasive surgery was associated with a reduction in mortality and an increase in good functional outcome, particularly when performed early after symptom onset [15]. The Dutch Intracerebral haemorrhage Surgery Trial (DIST) pilot study (NCT03608423) has recently demonstrated in 40 patients with ICH that minimally-invasive endoscopy-guided surgery within 8 h of symptom onset using the novel endoscopy-guided approach was feasible, safe, and technically effective for hematoma removal (Sondag, personal communication). At the time of our research, members of our team (CK, LS, FS) were preparing a phase 3 randomised clinical trial to evaluate effectiveness on functional outcome using early minimally-invasive endoscopy-guided surgery in patients with ICH (NCT05460793). Against this background we employed focus groups, interviews, and a survey to elicit [1] views of stakeholders on the new endoscopic device and (2) the quality of the (received) hospital care for ICH-patients.

### Selection procedure participants

We selected participants who are involved in hospital care for ICH and involved in the use of the new endoscopic device. Three stakeholder groups were identified: patients and relatives, healthcare professionals (HCPs), and policy experts. The number of participants that were determined for each method was guided by the concept of information power, which helps to establish how many participants are needed to acquire a reliable dataset [16]. Patients, relatives, and HCPs were approached by KW, LS and FS. LS and FS had a physician–patient relationship with the invited patients and relatives, or knew patients and relatives because they had participated in a previous studies related to ICH. HCPs were approached via the network of the neurologists involved in this research (CK, LS, FS), which spans over various ICH care institutions across the Netherlands. Policy experts were approached through networks of CK and MR. In the initial invitations, participants were assigned at random to one of the three methods. Participants that were invited for one method were not allowed to participate in other methods, so there were no cross-overs between methods. Subsequently, all potential participants were contacted via e-mail or by phone. After expressing their willingness to participate, they received an information letter and an informed consent form. In these documents, the aim, conditions, practical details, advantages, and disadvantages of participation were explained. All participants were given two weeks to consider participation, and the conditions of participation were again discussed or presented before each interview, focus group, or survey started. All participants gave written or verbal consent on audio tapes. The local certified ethics committee approved the research protocol.

### Data collection method 1: focus groups

We organised five 45-min focus groups, all planned on one evening. The first three focus groups were homogeneous: one for patients, one for HCPs, and one for policy experts. These three focus groups were conducted simultaneously. In these sessions, patients and relatives, HCPs, and policy experts convened in their own groups to discuss their views on the intervention with the new endoscopic device and the quality of hospital care. The participants were first placed in homogeneous groups to familiarize and become comfortable with people with a comparable perspective and make participants at ease. They were also placed in these groups to mirror the first round of interviews that was not primarily aimed at interaction but at constructing the different views of participants. The last two focus groups were also planned simultaneously, fifteen minutes after the first three focus groups. In these focus groups the patients and relatives, HCPs, and policy experts who participated in the first round of focus groups were mixed into two heterogeneous groups to discuss findings from the earlier homogeneous focus groups and to discuss how the different perspectives could be brought together. A number of four to twelve participants per focus groups is advised in literature, so we aimed to include six persons in the homogeneous focus groups, and nine persons in the ensuing heterogenous focus groups [17]. A minimum number of participants for qualitative research has been proposed of 12, so we aimed to include a higher number than that and tried to include a number that was comparable to the interviews [18]. Each focus group was led by one moderator, who probed for more in-depth answers if needed, and ensured that every participant was able to participate. All moderators were experienced in moderating group discussions and not in any way involved in ICH-care. Two moderators who participated in the first round moderated the second round. We used semi-structured focus groups protocols (see Appendix 1). All focus groups were audio-recorded and transcribed verbally. The audio tapes and transcripts were stored in accordance with European data safety requirements [19]. Due to the COVID-19 pandemic, the focus groups were organised online via a video call platform. The literature on online or ‘synchronous’ focus groups and interviews indicates that there can be some methodological specificalities to online data collection. It might improve the representation of certain groups, e.g. geographically dispersed people and people with disabilities, and it might be easier to generate richer data on sensitive topics. However, visual ques are harder to read [20–22]. Altogether, online focus groups might therefore be a useful way of data collection, especially for patients with ICH that encounter many mobility issues and social obstacles.

### Data collection method 2: interviews

For the interviews, we used fourth-generation evaluation methodology with some minor modifications [23–25]. All participants were interviewed twice: in the first round they were asked to share their views on the new endoscopic device and the quality of hospital care, and in the second round they were invited to respond to rival claims of other participants. We aimed to include a number that was comparable to the focus groups and above the minimum number of 12 participants that has been proposed [18]. All interviews were conducted by KW (3 years of interviewing experience). For both interview rounds, a semi-structured interview protocol was used (see Appendix 2). All opinions were processed and presented to other participants anonymously. Due to the COVID-19 pandemic, all interviews were taken via a video call platform, or by phone. As described above, online or synchronous data collection has distinctive features and might be a valuable method of data collection. All interviews were audio recorded and transcribed. The audio tapes and transcripts were stored in accordance with European data safety requirements [19].

### Data collection method 3: qualitative survey

The survey started with information about the study, then asked questions about characteristics of the person filling out the survey, including age, gender, education level, profession, and subsequently questions about improvements in the hospital treatment for ICH and the minimally invasive intervention with the new device. Patients and HCPs were asked to list unmet needs of patients and relevant outcome measures. The questions were closed, answer categories limited, but always followed by an opportunity to provide additional information in an open text field. We invited 78 participants, above the number of participants that proposed as a minimum in qualitative research (which is 12) and above the number of participants that is proposed for qualitative surveys (which is 25 to 50) [18, 26]. The survey was conducted via Castor, a digital survey platform. The data were stored in accordance with European data safety requirements [19].

### Analysis

#### Units of comparison

The three methods were compared on three outcomes: the number of themes, interaction, and the time-investment of the methods, which are described below.

#### Number and type of themes

The analysis of the number and type of themes was supported by Atlas.ti software (V.9) and performed by one author (KW) to increase consistency. Following the six steps of thematic analysis by Braun and Clarke [27], the analysis started with reading and coding the transcripts and survey answers. In later phases these codes were arranged and grouped into themes. This process was discussed with MT and RR to check the credibility of the codes and the themes to ensure consistency and minimise a possible bias. We compared the number of themes provided by each method, and determined what themes were constructed in only one of the methods. Counting themes may not be a conventional approach in qualitative research. However, we aim to provide insight into the breadth and thus comprehensiveness of information collected. Because the coding method was consistent along the three methods, we believe counting themes is an adequate unit of comparison.

#### Interaction

To assess interaction, we performed three analyses to determine: (i) what themes were addressed by multiple stakeholder groups, (ii) which participants interact with each other, and (iii) how participants interact. All these analyses were performed after the data were collected, and supported by Atlas.ti software (V.9). For the first analysis, we calculated the percentage of the themes that was addressed by two or three stakeholder groups in each data-collection method. For example, if both HCPs and policy experts addressed theme X, this theme was marked as ‘shared’, and consequently the percentage of shared themes of the total amount of themes could be calculated. For this comparison, all methods were taken into account, even though participants did not directly interact in surveys. For the other two analyses, we examined how participants interact. To this aim, we adapted coding schemes by Morgan & Hoffman [28] and Keyton [29], which can be applied on verbatim transcripts to code *how* people react. Our main aims for this analysis were to identify how extensive topics were addressed, and to identify whether stakeholders could reach forms of agreement while interacting. The codes as described by Morgan & Hoffman [28] were most applicable for these aims so we have used their code book, whereas Keyton [29] more concisely describes how different turns in an interaction can be coded, so we have adapted her coding process. The main change in the coding book that we made, is that we distinguished who interact from the ways how people interact, which was not clearly incorporated in Morgan & Hofmann’s code book (see Appendix 3). We coded who the speaker is and to whom this speaker addresses the message, which will be referred to as *turn*. When a moderator asked a question to participants, or vice versa, this is coded as a ‘moderator-participant’ turn, and when participants ask and answer questions to each other, these are coded as ‘turn between participants’. When a moderator presented views of other participants, and when participants reacted, these were separately coded as a ‘turn between moderator and participant where moderator presents view of others’. Besides *turns* we also coded how participants interact in the conversation, using the codes ‘question’, ‘answer’, ‘expansion: sharing new aspect of previous topic’, and ‘agreement’, which will be referred to as *acts*. Acts are mutually exclusive, but multiple acts could be assigned to one turn in a conversation and vice versa, for example when a participant gives an answer to a moderator and directly poses a question the moderator. KW performed the coding process. These analyses were discussed in-depth with MT and RR, to ensure consistency and minimise a possible bias.

#### Time-investment

We have operationalised time-investment as the absolute number of hours that all the involved researchers and moderators spent on preparing and performing data-collection and analysing the data. We chose not to analyse the investment from the perspective of the participants, because in this paper aims to compare participatory methods from the perspective of the person or organisation conducting them. Time-investment was based on the researchers’ (KW) agenda, supplemented by sources in the literature, to establish the average time that is needed to transcribe a one-hour audio-tape [30, 31]. Because the focus groups and interviews were conducted online, travelling time for researchers and moderators was not taken into account. The calculation of time-investment is set out in detail in Appendix 4.

#### Researcher characteristics and reflexivity

The research team partially consists of researchers developing qualitative evaluation methods (KW, MT, MR, RR), and partially of neurologists (CK, FS, LS). There was a doctor-patient relationship between FS and some of the participants. RR is an expert on interactive interviews.

## Results

### Participants

We invited 24 persons for the focus groups of whom 18 participated, 23 for the interviews of whom 17 participated, and 79 for the survey of whom 43 participated (see Table 1). In the focus groups, we had equal numbers of patients or relatives, HCPs, and policy makers: 6 participants in each group. The 17 participants in the interviews were patients (*n* = 7 and *n* = 3 in round 1 and 2), HCPs (*n* = 8 and *n* = 6 in round 1 and 2), and policy makers (*n* = 2 in both rounds). In the survey, we included 21 patients, 18 HCPs, and 4 policy makers. See Appendix 5 for an overview of the types and number of enrolled participants per data-collection methods that is completed with the total number of invited participants. The majority of interviews lasted an hour, but the duration varied from 30 to 90 min. All focus groups lasted 45 min.

**Table 1:** Number of participants in focus groups, interviews and the survey

Perspective	Stakeholder group	Focus group	Interview	Survey	Total
Patient and relative	Patients	4	3	11	18
	Relatives of patients	2	4	10	16
Healthcare professional	Neurologists	1	1	5	7
	Neurosurgeons	1	4	3	8
	Other relevant specialists (Radiologist, ICU-specialist)	2	2	4	8
	Nurses, other hospital personnel	2	1	6	9
Policy expert	Funding organisation representatives	2	0	3	5
	Policy makers	2	1	1	4
	Industrial partners	2	0	0	2
	Insurance/reimbursement	0	1	0	1
**Total**		**18**	**17**	**43**	**78**

#### Number and type of themes

**Fig. 1:**
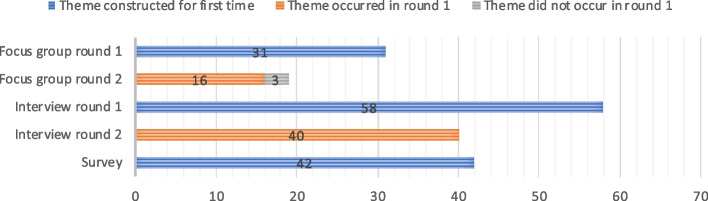
Number of themes constructed in three data collection methods

The number of themes varied between the different methods (see Fig. 1). In the explorative round 31 themes were constructed. In the interactive round 19 themes were constructed and 16 of these were also discussed in the first round, so 34 themes were constructed in all focus groups. The first interview round generated 58 themes, the second 40, and all themes in the second round of interviews had already been discussed in the first round. The survey generated 42 unique themes. An overview of all the themes generated can be found in Appendix 6.

All methods generated themes about research improvements for the innovation and care experiences in the hospital but there were some unique themes that specifically came up in one of the methods and not in the others. In the focus groups, four themes were constructed that were not generated in the other methods, two of which related to communication between HCPs and patients and relatives. In the interviews 19 themes were generated, that addressed various aspects of the assessment of the new treatment, such as the characteristics of the study population that ideally should undergo the new treatment in a subsequent research phase, and the informed consent procedure of that research phase. In the survey, 9 unique themes were present, ranging from ‘trust in HCPs’ to ‘outcome measure: feasibility’.

#### Interaction

##### Percentage of themes that is addressed by multiple groups of stakeholders

**Fig. 2:**
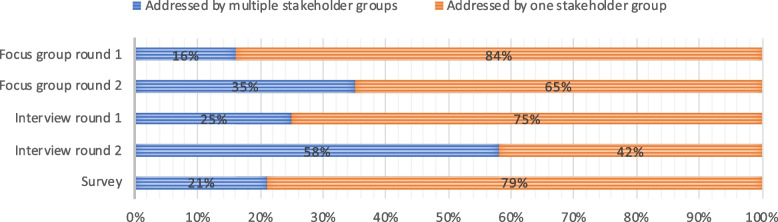
Percentage of themes addressed by multiple stakeholder groups in three data collection methods in round 1 (exploration) and round 2 (reaction)

Figure 2 shows that some themes were addressed by multiple stakeholder groups, whereas other themes were mentioned by one stakeholder group only. In the first explorative round of the focus groups, 16 percent of the themes was addressed by two or three stakeholder groups. In the second interactive round the percentage of themes addressed by multiple groups was 35 percent. In the first round of interviews the percentage of themes addressed by multiple groups was 25 percent in the first round and 58 percent in the second round. In the survey, 21 percent of the themes was addressed by multiple stakeholder groups, and these shared themes are all based on the numeric answers. None of the open-text box answers led to themes that were addressed by multiple stakeholders.

##### Which participants interact with each other: turns

**Fig. 3:**
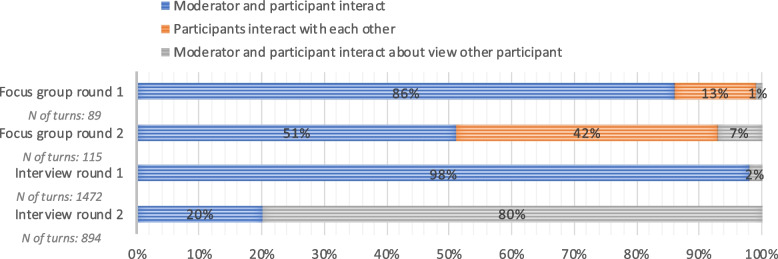
Which participants interact with each other: presented as percentage of total number of interactions

Figure 3 depicts which participants interacted with each other. In the first round of the focus group the moderators and participants primarily interacted, whereas in the second interactive round, participants more often interacted directly with each other. In the first interview round, 98% of the turns consisted of direct interaction between participants and the moderator and 2% of the turns consisted of interactions about other participants’ perspectives. In interview round 2, 20% of the turns consisted of direct interaction, and 80% percent of the turns entailed other participants’ opinions. The total number of turns in focus groups round one is 89, in round two 115. The total number of turns in interview round one is 1472 and in round two 894.

##### How participants interact: acts

**Fig. 4:**
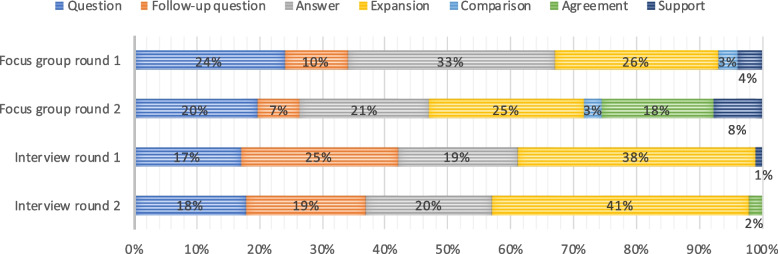
Form of interaction as percentage of total number of interactions

Figure 4 presents how participants and moderators interacted in each of the rounds in the focus groups and interviews. In the first round of the focus groups, 93% of the acts consisted of questions, follow-up questions, answers, and expansions on topics, whereas concrete expressions of agreement (18 percent) and support (8 percent) were more prevalent in the second round. In interview round one, 99% of the acts consisted of questions, follow-up questions, answers, and expansions upon previous answers. In round two this was 98%.

#### Time-investment

**Fig. 5:**
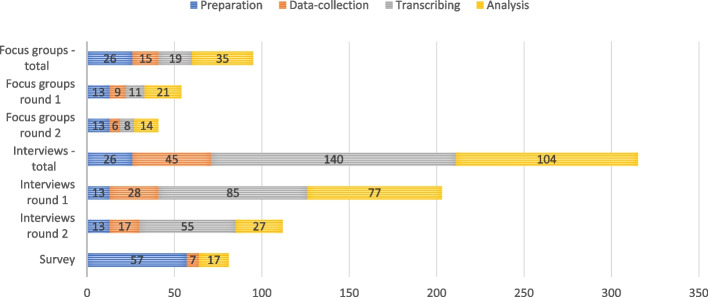
Accumulative number of hours worked by researchers

Figure 5 provides insight in the time-investment of each of the methods, operationalised as the number of hours that needs to be worked by all researchers. The preparation, data-collection, and analysis of the focus groups took 26, 15, and 54 h within in an overall time span of seven months. Of these 54 h of analysis, 19 consisted of transcribing interviews. The recruitment of participants for the interviews and setting a date took a preparation time that spanned over 5 months. The interviews took 26 h, 45 and 244 h for preparation, data-collection and analysis in a time span of 5 months and 10 months for the first and second round. Of these 244 h of analysis, 140 were dedicated to transcribing. The survey took 57 h to prepare and was completed over the course of nine months, as all questions had to be carefully integrated, checked, and piloted using a digital survey platform.

## Discussion

### Summary main findings

This comparison of focus groups, interviews and a survey in an empirical case study on minimally-invasive endoscopy-guided surgery in patients with ICH showed considerable differences in themes, interaction, and time-investment. In the focus groups relatively few themes were discussed, in the survey slightly more themes occurred, and in the interviews the largest number of themes was constructed. Many improvements for the assessment of the minimally invasive procedure were only discussed in the interviews, such as in- and exclusion criteria for the randomised clinical trial. We assume this is due to the extended time that can be dedicated to data collection, so that more detailed topics can be discussed in-depth. In other respects, there were no clear patterns of topics that occurred in specific methods. In terms of interaction, the focus groups where characterised by relatively much agreement and support. In the interviews agreement and support were seldomly expressed: nearly only questions and answers occurred. In terms of time-investment, the survey required the lowest total time investment: a total of 81 h, followed by focus groups with 96 h, and interviews required the highest time investment with a total of 315 h. There were no significant differences in time investment between the two rounds, neither for the focus groups nor for the interviews. The time allocated for preparation, data collection, and analysis was proportional to the number of interviews and focus groups conducted in each round.

Taking these results together, we can conclude that interviews generated the most useful data within the clinical case because they generated the highest number of relevant themes. They did however require a high-time investment. Focus groups appeared to be a technique that can better be employed to generate agreement and support instead of generating many relevant themes. Surveys do not generate the most comprehensive overview of themes, but can be useful if researchers want to map themes in a quick way without asking follow-up questions. It is important to be aware that we analysed these differences in the context of medical device evaluation, where the aim of participatory methods is to inform the research or development process, rather than merely describing stakeholder views.

### Comparison literature

Our results are in agreement with other studies that show that focus groups generate fewer themes compared to interviews, and that focus groups are relatively time efficient [32–34]. Other studies, however, report that focus groups and interviews generate a comparable large number of themes, whereas our results clearly show that interviews generate more themes [10, 35–37]. The discrepancy might be explained by the large number of focus groups that were held and the number of participants that participated in these latter studies. Namey et al. [35] for example assigned 310 participants to 40 focus groups and 40 participants to interviews. Because these authors include such high numbers in focus groups, they also state that focus groups require a lower time-investment. Based on our findings and the findings in the literature, we can assume that with equal inclusion numbers, focus groups take less time at the expense of generating less data.

### Strengths and limitations

The major strength of our study is that we compared three participatory methods in one single empirical case study on the development and evaluation of a medical device. With rigorous control of many contextual and methodological factors we can conclude that the differences in the three outcomes of this study are mainly caused by the characteristics of the data collection methods. Another strength is that we have embedded the comparison in an actual participatory assessment of an innovative minimally-invasive surgical treatment, increasing the ecological validity of the results. Patients and their relatives were involved, they interacted with different physicians and policy makers, and the findings were used in later assessments of the new procedure. Therefore, the results of this study are representative for interactions between these groups. The insights have also informed subsequent quantitative studies on the surgical treatment. Albeit our study is focussed on medical device evaluation, it also has consequences for comparisons of qualitative methods in a broader perspective, because we have adopted conventional focus group, interview and survey methodologies. Our results can therefore be compared with those from related studies outside the domain of medical device evaluation. Finally, we performed an analysis of interaction. The analysis of interaction is rarely done, probably because it is laborious. However, this analysis is crucial because interaction between participants and between participants and moderator(s) is one of the primary aims of focus groups and interviews. By showing who interacts with whom and how, we offer insights in the processes of these methods, which can be of use in the selection of one of these methods by others.

Some limitations should also be addressed. First, there are still some variations in the three research designs that could have influenced the outcomes. Different moderators and stakeholders were involved in each method, and their specific characteristics could have affected the results. Using different moderators and participants was a considerate choice, however, since the use of the same moderator or participants in all methods would have influenced the outcomes, as they would have taken their experiences from one activity to the other. Second, a substantial proportion of themes was found in only two or one of the methods, which indicates that none of the methods generated a complete set of data. This implies that the concept of information power might not have been the most suitable technique to determine the number of participants. Saturation might be a better point to close the data collection phase, especially when a diverse range of participants is included, such as in this study. Third, in the survey a relatively low number of participants were enrolled. The data may therefore not be representative of surveys with higher inclusion numbers. Yet, the survey in this study was constructed for qualitative research aims and not intended to produce quantitatively significant results. Fourth, the data of all three methods were collected digitally, because the data-collection was performed during the COVID-19 pandemic. Therefore, the results may not be directly applicable to face-to-face interactions. As work is increasingly being digitalised and organised remotely, it is likely that in the future qualitative data more often are being collected by means of video consulting applications.

### Implications

Our results help researchers and innovators to choose a method that is most suitable for their research aim, both in- and outside the domain of medical device evaluation. First we will place the methodological findings in the clinical context in which we worked. Next, we will discuss contextual factors that are relevant for medical device evaluation in general.

As described in the methods section, three methods were compared against the background of the design of a phase 3 randomised clinical trial to evaluate early minimally-invasive endoscopy-guided surgery in patients with ICH (NCT05460793). The clinical results of our research were used to inform different aspects of this randomised clinical trial, such as the selection of relevant outcome variables and the process of acquiring informed consent in this acute intervention trial. The results were also used to see how hospital care for ICH could be improved in general. All methods generated relevant insights in the context of this study, but interviews generated the most comprehensive body of relevant results for the trial and were thus most useful in the specific clinical context.

As described, there are some implications that are relevant for all types of researchers or innovators that want to employ participatory methods. Elucidating needs of stakeholders might especially be relevant in early development stages since they can be translated in the further development and evaluation of a device. It is thus relevant to have a comprehensive, in-depth assessment of these needs and interviews appear to be most appropriate for that aim. If in later stages more specific development issues need to be resolved, stakeholders could be brought together in a small focus group to make a joint decision. Since financial investments are required for participatory evaluations, the question arises whether the benefits live up to these investments. In the literature, stakeholder involvement is generally considered to be beneficial, but an important caveat is that low budgets and small-scale involvements can lead to flawed stakeholder representation and reproducing existing power differences [38]. We strongly recommend to invest in participatory development and evaluation as this is likely to be highly beneficial, because the perspectives of stakeholders are embedded in the innovation process.

Furthermore, we posit the existence of overarching methodological implications pertinent to participatory research at large, and delineate some considerations. The body of results generated by focus groups was not as comprehensive as in the interviews. This is possibly due to the social dynamic that is at play between participants and the limited time that each individual speaker is talking, which makes expansive questioning by a moderator on all relevant topics hard to realize. This implies that for comprehensively answering a research question, focus groups are not the most suitable method. Nevertheless, topics can be discussed and resolved relatively quickly in a focus group if the participants are more or less on the same page, which implies that focus groups can be a useful decision-making technique. Interviews can best be used for in-depth analyses of different perspectives, to generate much data, and therefore comprehensively answer a research question. Researchers or innovators may shorten the time-investment of interviews by speeding up the analysis, for example by not transcribing full interviews but writing summaries directly after an interview. Surveys might be suitable if researchers or innovators want to carry out a fast exploration of themes, and do not require interaction or an in-depth explication of topics. Qualitative surveys lack the option for inductive questioning, so they are not suitable if researchers want to apply an open research design. Methods presented in this paper could also be combined, or integrated in a stepwise manner in a research of development process. A researcher might first use a survey to explore multiple stakeholders’ wishes, and subsequently arrange a round of interactive focus groups when fast and definite decision needs to be made. An evaluation trajectory of medical devices is hard to plan in advance and unforeseen issues might arise, and these issues might demand specific participatory methods.

## Conclusion

Focus groups, interviews, and surveys have clear methodological differences and provide different results within the context of medical device evaluation. Focus groups can best be used to bring views together, but do not enable a comprehensive analysis. Interviews enable an in-depth analysis of stakeholder views and can best be used to comprehensively answer an explorative research question, but are time-intensive. Surveys can be used for a rapid exploration of perspectives. Researchers should account for these methodological differences and select the method that serves their research aim.

### Supplementary Information

**Supplementary Material 1.**

**Supplementary Material 2.**

**Supplementary Material 3.**

**Supplementary Material 4.**

**Supplementary Material 5.**

**Supplementary Material 6.**

## Data Availability

No data are available. All relevant data are presented in this study.
