# Supplementary material for: Participatory methods used in the evaluation of medical devices: a comparison of focus groups, interviews, and a survey
Source: BMC Health Serv Res. 2024 Apr 12;24:462. doi: 10.1186/s12913-024-10887-3 (PMC11015660; doi:10.1186/s12913-024-10887-3)
Supplement: Supplementary file 1 — Supplementary Material 1. [file 12913_2024_10887_MOESM1_ESM.docx]

Supplementary file 1. Focus group protocols

*This file is submitted in accordance with the SAGE author guidelines for supplemental material*

**Patients and relatives (round 1)**

1. Introduction 15 min

- Introducing the moderator
- Purpose of the research: to identify improvements in hospital care for brain hemorrhages. This will enable care and further research to be tailored to the needs of important persons who are involved in the care. We will also conduct research through other methods: an interview study and a questionnaire. This will enable us to investigate the differences between these methods.
- Due to time constraints, there may not be enough time to cover all topics. However, everything that is said is relevant and will be taken into account during the analysis?

*To each participant individually:*

- Can you briefly introduce yourself?

1. What can be improved? 25 min

*To each participant individually:*

- What are areas for improvement in the hospital care for brain hemorrhages?

*To the group:*

- Does anyone want to respond to these areas for improvement?
- Who recognizes these points? Can you explain why?
- Who does not recognize these points? Can you explain why?

*To the group:*

Which improvement do we consider the most important to discuss further? (The number will depend on the available time).

1. What are the solutions? 15 min

*To each participant individually:*

- What solutions do you have in mind?
- It may help to think about who can provide a solution, and how that solution can work.

*To the group:*

- Does anyone want to respond to these solutions?
- What is the benefit of this solution?
- What stands in the way of this solution?

To the group:

- Which solutions do you consider the best, and why?

**Healthcare Professionals (Round 1)**

1. Introduction 15 min

- Introducing the moderator
- Purpose of the research: to identify improvements in hospital care for brain hemorrhages. This will enable care and further research to be tailored to the needs of important persons who are involved in the care. We will also conduct research through other methods: an interview study and a questionnaire. This will enable us to investigate the differences between these methods.
- Due to time constraints, there may not be enough time to cover all topics. However, everything that is said is relevant and will be taken into account during the analysis.

*To each participant individually:*

- Can you briefly introduce yourself?

1. What can be improved? 25 min

*To each participant individually:*

- What are areas for improvement in the hospital care for brain hemorrhages?

*To the group:*

- Does anyone want to respond to these areas for improvement?
- Who recognizes these points? Can you explain why?
- Who does not recognize these points? Can you explain why?

*To the group:*

- Which improvement do we consider the most important to discuss further? (The number will depend on the available time).

1. What are the solutions? 15 min

*To each participant individually:*

- What solutions do you have in mind?
- It may help to think about who can provide a solution, and how that solution can work.

*To the group:*

- Does anyone want to respond to these solutions?
- What is the benefit of this solution?
- What stands in the way of this solution?

*To the group:*

- Which solutions do you consider the best, and why?

**Policy Experts (Round 1)**

1. Introduction 15 min

- Introducing the moderator
- Purpose of the research: to identify improvements in hospital care for brain hemorrhages. This will enable care and further research to be tailored to the needs of important persons who are involved in the care. We will also conduct research through other methods: an interview study and a questionnaire. This will enable us to investigate the differences between these methods.
- Due to time constraints, everything that is said is relevant and will be taken into account during the analysis.

*To each participant individually:*

- Can you briefly introduce yourself?

1. Introduction of stroke case
2. Stroke is a common problem with 6617 hospital admissions per year. Unfortunately, there are currently few effective treatment options.
3. One option is surgical removal of the stroke with a specialized device made for this purpose, the Artemis Neuro Evacuation Device made by Penumbra. Two representatives from this company are also present at the discussion.
4. Some studies have been done on the effectiveness of such removal. These studies were found to be ineffective, and the assumption is that the reason is because the surgeries were done several days after the onset of the stroke.
5. The current hypothesis is that removal of the stroke can be effective if it is done within 8 hours of the stroke. A pilot study is now being done to assess its safety.
6. If successful, a large RCT will be conducted in multiple centers throughout the Netherlands.
7. Evaluation of case and new procedure, 20 min

*To each participant individually:*

- What questions come to mind for you?
- What opportunities or threats do you see when you hear about this case?
- When are you convinced of the added value of this new procedure?

*To the group:*

- What do you think of the ways in which new procedures or technologies are evaluated?

1. Policy improvements, 20 min

*To each participant individually:*

- When is an adequate evaluation procedure in place?
- What needs to be improved compared to the current evaluation procedure?

*To the group:*

- What do you think of these improvements?
- Do you recognize these improvements in your own work?

**Heterogeneous groups (round 2)**

1. Proposal, 5 min

- Introduction of moderator
- Can you briefly introduce yourself?

1. Presentation of improvement points, 15 min

*To patients:*

- Can you describe the improvement points discussed in the previous focus group?

*To doctors:*

- Can you describe the improvement points discussed in the previous focus group?

*To policymakers:*

- Can you describe the improvement points discussed in the previous focus group?

1. Relations between improvement points, 15 min

*To the group:*

- How do improvement points coincide?
- How do these improvement points not coincide? And why is that?
- Which improvement points do we want to discuss?

1. Solutions, 15 min

*To the group:*

- We have just chosen 1 problem, what can the solutions be?
- Who can contribute to those solutions?
- What is needed for that solution?

1. Conclusion, 10 min

*To the group:*

- Thank you for your participation.
- What did you think of the focus group?
- What could have been done better in the process?
- A summary will be prepared based on the focus group. It will be sent to everyone so that you can also respond to it.
