# Supplementary material for: Participatory methods used in the evaluation of medical devices: a comparison of focus groups, interviews, and a survey
Source: BMC Health Serv Res. 2024 Apr 12;24:462. doi: 10.1186/s12913-024-10887-3 (PMC11015660; doi:10.1186/s12913-024-10887-3)
Supplement: Supplementary file 2 — Supplementary Material 2. [file 12913_2024_10887_MOESM2_ESM.docx]

Supplementary file 2. Interview protocols
*This file is submitted in accordance with the SAGE author guidelines for supplemental material*

**Patients (round 1)**

Thank you for agreeing to participate in our study. I will briefly explain the purpose of this study again. I work as a researcher in the operating department at Radboudumc. Together with researchers from the neurology department, we are conducting research on the experiences of people who have had a stroke. We are interested in what went well in the hospital treatment and where you think improvements could be made. Finally, we would like to discuss the opinions of other participants in our study. These are different people involved in stroke care, such as doctors, nurses, and policymakers. We present their opinions anonymously and then ask for your reaction.

You have received an information letter and a consent form from us. If you agree, we will record this interview. The information from the interview will then be processed anonymously. We are required to securely store the transcribed interview for 15 years. You can stop participating in the study at any time without giving a reason. You can always ask me questions during the study.

**Background**

Can you tell us about yourself?

*Clarifying Questions*

How old are you?

What do you do in your daily life?

What do you think are important things to do in your life?

**Stroke Experience**

Can you tell us how you are doing in general now?

*Clarifying Questions*

What is going well in your life now?

What could be better in your life now?

What do you consider valuable activities to do now?

How has your life changed since the stroke compared to before?

**Treatment process status quo**

Can you describe your treatment process?

*Clarifying Questions*

What treatment did you receive?

How did you experience the treatment?

You mentioned at the beginning of the conversation how you are doing now.

How did the previous treatment contribute to how you are doing now?

**Treatment process improvements**

What went well in your treatment process?

*Clarifying Questions*

What could be improved in your treatment process?

How could that be improved in the treatment?

You mentioned some valuable activities to do at the beginning of the conversation.

What are the areas of improvement in the treatment so that you can do those things more?

How could that be improved in the treatment?

**Case: A New Innovation**

Currently, there are few effective treatment options. A new treatment option is the surgical removal of the stroke with a special device made for this purpose. Some studies have been done on the effectiveness of such removal, but they have been found to be ineffective. Researchers think that these studies were ineffective because the operations were done too late after the onset of the stroke.

The current hypothesis is that removal of the stroke can be effective if it is done within 8 hours of the onset of the stroke. Currently, research is being done on the safety of this operation.

**Questions**

What are the first ideas or feelings that come to mind when you hear about this case?

Under what circumstances would you have wanted to participate in this study?

What outcomes should this innovation be judged on?

If time allows: Consent. People with a stroke are often unconscious, so they cannot give consent to participate in such research. What do you think is the best way to obtain consent?

*Clarifying questions*

What are the reasons why you would participate?

What are the reasons why you would not participate?

For example, whether the innovation ultimately makes people healthier or ultimately cheaper?

Refer to the patient's quality of life above.

What do you think of consent through family members?

What do you think of retrospective consent: the new procedure is performed, and then the patient is asked if the findings can be used when conscious.

Those were the questions we wanted to ask you.
Do you want to add anything else?
Have we missed any important questions?
Do you have any questions?

**Relatives (round 1)**

Thank you for participating in our research. Let me briefly explain the purpose of this study. I work as a researcher in the department of operating rooms at Radboudumc. Together with researchers from the neurology department, we are conducting research on the experiences of people involved in a brain hemorrhage. We are curious about what went well in the treatment of your loved one in the hospital and where you think there is room for improvement. Finally, we want to discuss the opinions of other participants in our research with you. These are different people involved in the care for brain hemorrhages, such as doctors, nurses, and policymakers. We present their opinions anonymously and then ask for your response.

You have received an information sheet and consent form from us. If you agree, we will record this interview. We will then process the information from the interview anonymously. We are obligated to securely store the written interview for 15 years. You can stop participating in the research at any time without giving a reason. You can always ask me questions during the research.

Do you have any questions about the interview or the research?

**Background**
Can you first tell us something about yourself?

*Clarifying questions*How old are you?
What do you do in your daily life?
What is your relationship with the loved one?

Can you tell us something about your loved one (the patient)?

*Clarifying questions*
How old is he/she?
What did he/she do in daily life?
What were important things for him/her to do in life?

**Experience of Brain Hemorrhage**
Can you tell us how your loved one is doing now in general?

*Clarifying questions*
What is going well now?
What can be improved?
What does your loved one consider valuable activities to do now?
Compare that to life before the brain hemorrhage, how has it changed?

**Treatment Trajectory Status Quo**
Can you describe the treatment trajectory?

*Clarifying questions*
What treatment did your loved one receive?
How did your loved one experience the treatment?
At the beginning of the conversation, you mentioned how your loved one is doing now. How did the past treatment contribute to how your loved one is doing now?

**Treatment Trajectory Improvements**What went well in the treatment trajectory?

*Clarifying questions*
What could have been better in the treatment trajectory?
How could that be improved in the treatment?
You mentioned some valuable activities at the beginning of the conversation.
What are areas for improvement in the treatment so that your loved one can do those things more?
How could that be improved in the treatment?

**As the role of the loved one**How are you doing now?

*Clarifying questions*
How did the current/past treatment contribute to how you are doing now as the loved one?
What could be improved in the treatment for you as the loved one?
How could that be improved?

**Case: A new innovation**
Currently, there are few effective treatment options. A new treatment option is the surgical removal of the brain hemorrhage with a special device designed for this purpose. Several studies have been done on the effectiveness of such removal, but they were found to be ineffective. Researchers believe that these studies were ineffective because the surgeries were performed too late after the onset of the hemorrhage.

The current hypothesis is that removal of the brain hemorrhage can be effective if done within 8 hours of the onset of the hemorrhage. Currently, research is being conducted on the safety of this operation.

**Questions**
What are the first ideas or feelings that come to mind when you hear about this case?
Under what circumstances would you have wanted to participate in this research?

What outcomes should this innovation be evaluated on? If there is enough time: Consent. People with a brain hemorrhage are often unconscious, so they cannot give consent to participate in such research. How do you think consent should be obtained?

*Clarifying questions*
What are reasons why you would participate?

What are reasons why you would not participate? For example, whether the innovation ultimately makes people healthier or ultimately cheaper?

Refer to the patient's quality of life above.

What do you think of consent through family members?

What do you think of retrospective consent: where the new procedure is performed and the patient is asked when conscious whether the findings may be used.

Those were the questions we wanted to ask you.
Do you want to add anything else?
Have we missed any important questions?
Do you have any questions?

**Healthcare professionals (round 1)**

Thank you for agreeing to participate in our research. I will briefly explain again what the purpose of this study is. I work as a researcher in the operating room department at Radboudumc. Together with researchers from the neurology department, we are conducting research on the experiences of people involved in a cerebral hemorrhage. We are interested in what you think is going well in the hospital treatment, and where you think improvements are possible. Finally, we want to discuss the opinions of other participants in our study. These are different people involved in the care of cerebral hemorrhage, such as patients, other doctors and nurses, and policymakers. We present their opinions anonymously and then ask for your reaction.

You have received an information letter and a consent form from us. If you agree, we will record this interview. We will then process the information from the interview anonymously. We are obliged to keep the written interview securely for 15 years. You can stop participating in the study at any time and do not need to give a reason. You can always ask me questions during the study.

Do you have any questions about the interview or the study at this time?

Background

Can you tell us something about yourself?

*Clarifying questions*

How old are you?

What is your specialty?

What are your daily activities in the hospital?

**Experience with cerebral hemorrhage**

Can you describe the treatment trajectories in the hospital that you are involved in?

In which treatment are you most involved?

Can you name several positive and negative aspects for each treatment?

**Treatment trajectory Improvements**

Can you name three things that could be improved in the hospital treatment?
How can these improvements be made?

Case: a new innovation At present, there are few effective treatment options for cerebral hemorrhages. A new treatment option is surgical removal of the cerebral hemorrhage. Some studies have been done on the effectiveness of such a removal. These studies found the treatment to be ineffective. Researchers believe that these studies were ineffective because the surgeries were performed too late, namely 72 hours after the onset of the cerebral hemorrhage.

The current hypothesis is that removal of the cerebral hemorrhage can be effective if performed within 8 hours after the onset of the hemorrhage. Research is currently being conducted on the safety of this surgery in the Dutch-ICH trial, a multicenter trial running throughout the Netherlands. If this goes well, a phase III randomized controlled trial (RCT) will follow.

**Questions**

What are the first ideas or feelings that come to mind when you hear about this case?
What do you think about this research?

If familiar with research: What could be improved in this research? What outcome measures should be chosen in this research?

If there is enough time: Consent. People with cerebral hemorrhage are often unconscious and therefore cannot give consent to participate in such research. What do you think about obtaining consent? What are the positive aspects of this research? What are the negative effects of this research? How can this be improved?

Those were the questions we wanted to ask you.
Do you want to add anything else?
Have we missed any important questions?
Do you have any questions?

**Policy experts (round 1)**

Thank you for participating in our research. I will briefly explain again what the purpose of this study is. I work as a researcher in the operating rooms department at Radboudumc. Together with researchers from the neurology department, we are conducting research on the experiences of people involved in a brain hemorrhage. We are interested in what you think is going well in the hospital treatment, and where you think there is room for improvement. Finally, we want to discuss the opinions of other participants in our study. These are various people involved in the care for brain hemorrhages, such as patients, doctors, nurses, and other policy makers. We present their opinions anonymously and ask for your reaction.

You have received an information letter and a consent form from us. If you agree, we will record this interview. The information from the interview will be processed anonymously. We are required to securely keep the written interview for 15 years. You can stop participating in the research at any time without having to give a reason. You can ask me any questions at any time.

Do you have any questions about the interview or the study?

Background
Can you tell us something about yourself?

*Clarifying questions*

How old are you?

In what way are you involved in the policy for care or research into brain hemorrhages?

**Brain Hemorrhage Experience**

Can you describe the treatment processes in the hospital that you are involved in?

Can you name some positive and negative aspects for each treatment?

Which treatment are you most involved in?

**Treatment trajectory Improvements**

Can you name three things that can be improved in the hospital treatment? How can this be improved?

**Policy Improvements**

Can you name three things that can be improved in your daily activities? How can this be improved?

Case: A New Innovation Currently, there are few effective treatment options for brain hemorrhages. A new treatment option is the surgical removal of the brain hemorrhage. Some studies have been done on the effectiveness of such a removal, but they were found to be ineffective. Researchers believe that these studies were ineffective because the surgeries were done too late after the hemorrhage had occurred.

The current hypothesis is that removal of the brain hemorrhage can be effective if done within 8 hours of the onset of the hemorrhage. Currently, research is being conducted on the safety of this operation in the Dutch-ICH trial, a multicenter trial that is being conducted throughout the Netherlands. If successful, a phase III randomized controlled trial (RCT) will follow.

**Questions**

What are the first ideas or feelings that come to mind when you hear about this case? What do you think about this research?

If familiar with research: What can be improved in this research? What outcome measures should be chosen in this research?

If time allows: Consent. People with a brain hemorrhage are often unconscious, which means they cannot give consent to participate in such research. What do you think is the best way to obtain consent?

Those were the questions we wanted to ask you.
Do you want to add anything else?
Have we missed any important questions?
Do you have any questions?

**All stakeholder groups (round 2)**

All participants were asked to share their thoughts about the following document that was send to them a week before the interview:

RESULTS OF INTERACTIVE EVALUATION ROUND 1

Improvements in Hospital Care for Brain Hemorrhages and Innovations in a Study Please find enclosed an overview of the findings from the first round of the interview study. The aim of this study is to explore with patients, doctors, and other important stakeholders what is going well and what can be improved in hospital care for brain hemorrhages (or intracerebral hemorrhages, further referred to as 'ICH' in this document). In addition, the question is how a large-scale study of an innovative operation can meet the needs of you and the other participants. This document contains an inventory of all the improvements mentioned by you and other participants. It will therefore also include topics that have not yet been discussed with you. In addition to this document, we have made a video summarizing the findings, so you can also watch the video. We would like to ask you to read through this document or watch the video before the interview takes place. We also request you to consider which points you agree or disagree with, and which things you find important.

Participants
8 healthcare professionals
7 patients or their relatives
2 policy experts

I. Improvements in hospital care

**Communication**

1. Communication between healthcare providers such as neurologists, intensive care physicians, radiologists, and nurses within one hospital can be improved according to various participants. Communication can be more extensive, more regular, and more often about ethical questions. An example of such a question is whether treatment can still lead to a valuable life. This is desirable because new healthcare providers sometimes offer care that does not fit the wishes, life, and health condition of patients.
2. According to several participants, communication between healthcare providers and patients and their relatives could be improved. The following points emerged in the interviews.
   a. Many participating patients and relatives experience that communication is not frequent enough. For example, many patients and relatives miss closing discussions after the care trajectory to discuss the care received.
   b. Communication can be done with more empathy towards the difficult situation for the patient and their relatives. For several patients and relatives, it is difficult not to receive any perspective for improvement. Offering a glimmer of hope would create a more positive feeling in the care trajectory.
   c. According to various participants, the language used could better suit the knowledge of patients and their relatives. Medical jargon is often used in communication.
   d. Conversations could be offered more actively. Patients or relatives often have to proactively request a conversation or fixed communication moments.
   e. Some participants feel that healthcare workers talk too often only with the patient. For important conversations, several participants would like the family to be involved.
   f. Certain topics can be discussed more frequently. These include:
   - 1. Treatment goals. These are sometimes unclear or poorly discussed according to various participants. Also, the patient's life before the hemorrhage is sometimes not discussed, leading to the wrong care being provided.
     2. ii. Psychological and social support. The psychological impact is often greater than expected for patients and their relatives. According to participants, this topic can be discussed earlier and in more detail.
3. Communication between hospital healthcare providers and healthcare institutions that take over care after hospitalization can be improved. Patients and relatives experience many problems during the handover. For example, they often have to repeat their medical history or treatment goals, leading to confusion and errors.

**Perspective on aftercare**
Many patients and their loved ones in the hospital have a need for information about social and psychological aftercare. The need for information about psychological and social support is evident, as many patients and their loved ones have a need for it in the hospital. Participants noted that patients and their loved ones are often not sufficiently informed about peer support through patient organizations or possible "buddy projects" that bring patients or their loved ones into contact with each other.

**Logistic problems**

Sometimes logistic problems arise in facilitating good tools, transportation or appropriate accommodation for the patient. Tools that were missing included a shower stretcher so the patient could not shower and fall protection in a bed to prevent the patient from falling out of bed. Problems with the ambulance arose in two cases during transportation. In one case, transportation from the hospital to the rehabilitation center was not arranged. Finally, the spaces on the nursing ward are often experienced as crowded and small.

II. Study

Currently, a large-scale study is being set up to investigate whether surgery with a minimally invasive device works better than the current treatment plan, which often does not involve surgery. The interviews discussed how the study can be improved. Part one is about what should be measured in the study according to the interviewees: the outcome measures. Part two is about the people who can best participate in the study within the study group: the study population. Part three is about how consent is obtained for participation in the study.

**Outcome measures**

1. Outcome measures are the things that are measured in a study. To assess whether a treatment works well, important outcomes need to be determined according to patients, loved ones, healthcare workers, and other experts. A policy expert who participated in the interviews indicates that there should always be three to five patient-relevant outcome measures: these are outcomes that are important for patients' lives.
2. Effectiveness of treatment, measured in: a) Survival: the percentage of people who survive the bleeding. b) Functioning, whereby the Modified Rankin Scale for Neurologic Disability (mRS score) is most commonly mentioned. This is a score that indicates the degree of disability. There is disagreement among participants about the outcome that should be taken as the minimum positive outcome. Some believe that score 2, a mild handicap, is the minimum outcome that should be achieved with treatment. Others believe that score 3, a moderate handicap, is a sufficient outcome for treatment. These are the mRS scores:
3. No symptoms.
4. No significant handicap despite the presence of symptoms; can perform all daily activities and tasks.
5. Mild handicap; unable to perform all previous activities, but can manage personal affairs without assistance.
6. Moderate handicap; needs some help, but can walk without assistance.
7. Moderately severe handicap; unable to walk without assistance and provide for one's own personal care.
8. Severe handicap; bedridden, incontinent, and requires constant care and attention.
9. Safety, measured in:
   a) The occurrence of a harmful event, referred to as "adverse events" and/or "expected adverse events" in jargon. For example, the percentage of people who experience bleeding after the surgery. What constitutes an "adverse event" or "expected adverse events" has not yet been extensively discussed.
10. The third important outcome measure is the quality of life as experienced by the patient and/or family themselves. The quality of life should then be measured through questionnaires before and/or after treatment.

a) These questionnaires should be completed by the patient themselves, to assess whether the quality of life has improved from the patient's perspective as a result of the treatment.
b) Relevant family members or close associates can also complete the questionnaire. Some participants suggest that patients themselves may find it difficult to perceive, for example, if they are becoming unhappy, whereas those close to them may be better able to notice such changes.
c) Participants identify a number of factors that are important for quality of life, including: preservation of speech and language processing, independence, memory, overstimulation, fatigue, stress, tension, anger, loneliness, relationship problems, independence, and prevention of delirium.
d) What patients consider to be a valuable life can change due to the bleeding or care trajectory. Some participants see this as a limitation of the questionnaire, as it can then be difficult to determine which questionnaire should be used to assess quality of life. Not all participants consider quality of life essential to be taken into account. According to some, measuring the effectiveness and functionality of treatment is sufficient in a large comparative study.

1. The duration of the care trajectory is also mentioned. According to the participants, this can be measured as:
   a) Length of stay in the hospital.
   b) Length of stay in the intensive care unit.
   c) Rehabilitation period. This outcome measure is seen by a policy expert as non-crucial and does not need to be taken into account in a large comparative study.
2. Cost-effectiveness is also mentioned as an outcome measure. In order to offer a treatment, the treatment must work well but not cost too much. If a treatment is too expensive, other care cannot be delivered. This outcome measure is seen by a policy expert as non-crucial and does not need to be taken into account in a large comparative study. Another expert indicates that proving cost-effectiveness alone is not enough to get care insured. The innovation will only be reimbursed by an insurer if the total costs of treating ICH do not increase.

**Study population**

According to the interviewees, it is important to involve the right people in the research. Therefore, they were asked about the ideal group of participants. The group of people who eventually participate in the research is called the study population. Various participants were consulted about the characteristics that this group of people should have in order to participate in the study. Some participants suggest that a study population should be selected where positive outcomes are expected. This way, it can be demonstrated first that the surgery works in an ideal group of participants, and then follow-up studies can be conducted with other participants. If too many different people participate in the study, the outcomes may not be positive because many people with poor outcomes are included. Some participants also suggest that a pragmatic approach should be chosen, so that enough people can participate within a reasonable time period. Inclusion criteria The inclusion criteria are the conditions that people must meet in order to participate in the surgery in the research.

**Inclusion Criteria**
The inclusion criteria are the conditions that people must meet in order to participate in the operation in the study.

1. The volume of the bleeding has been discussed extensively with healthcare professionals. According to some, a volume of 10 ml is a good minimum. Other participants believe that 15 to 20 ml is a better minimum because surgery is more meaningful due to increased mass effect and a greater inflammatory response. In relatively young people, volume is particularly important because the damage from bleeding can be particularly severe in them.
2. The degree of disability is another criterion. The degree of disability is determined when someone enters the hospital. Several methods have been suggested for measuring this: a) The clinical frailty score: This score indicates how "fragile" or "weak" someone is. This is determined by a specialist who makes this assessment based on observations and the patient's medical history. If someone is stronger, they are more likely to have a successful operation. b) The National Institutes of Health Stroke Scale: NIHSS. This score indicates the severity of a stroke or bleeding. A specialist determines a score based on observations. c) The Glasgow Coma Scale: This score indicates the degree to which someone is conscious. Again, a specialist determines a score based on observations.
3. Timing: According to most participants, 8 hours after the onset of the first symptoms is a good minimum. Others think that 12 hours is also a good minimum in order to involve more participants more quickly. They believe that waiting four hours longer will not make much difference in preventing mass effect of bleeding or slowing down inflammation.
4. Age: According to some participants, 80 years is an upper limit. Others indicate that the clinical frailty score, as described above, is a good substitute for age.

**Exclusion Criteria**
These are factors that determine that people should not participate in the study. These criteria are:

1. High blood pressure;
2. Use of blood thinners/anticoagulants;
3. Endocarditis, an inflammation of the heart that can later lead to bleeding;
4. Cerebral amyloid angiopathy, a neurological disorder that causes blood vessels in the brain to rupture more easily;
5. A brain tumor.

**Special Attention Areas**
Discussions with various healthcare providers have been held on cases where surgery would lead to significant improvements in treatment.

1. With deep-seated bleeding, many participants expect the most benefit because these are areas that cannot be reached with a regular operation. These include: a) Basal ganglia. These are important areas that provide many different functions. The thalamus is often mentioned separately; this is an important area for information processing and motor skills. b) Corticospinal tracts. These deep structures have a lot of influence on motor skills.
2. Bleeding in the brain lobes (lobar bleeding) is also mentioned as an area where the benefit can be great. The frontal lobe is specifically mentioned as an important area. This area is important for many different brain functions, such as planning and problem-solving.
3. Bleeding in the posterior fossa, as this area is difficult to reach with other equipment.
4. Bleeding in the ventricles, which are the result of other bleedings: the 'secondary intraventricular bleedings'. The ventricles are pathways through which fluid is delivered to the brain. The ventricles are deep, and the operation may be a solution here.

There are locations in the brain where participants prefer not to have surgery, as the benefits of an operation in this area may be low. These are:

1. The dominant hemisphere. This brain hemisphere is involved in speech and language, and operating here can cause damage.
2. The eloquent areas. These areas are important for speech, language, motor function, sensation, and vision. Surgery in this area can also cause damage.
3. In the case of arteriovenous malformation (AVM), a defect in the blood vessel system. Some participants indicate that surgery there is worthwhile, while others indicate that surgery is not helpful.

**Consent**There are two ways in which people can be asked for consent to participate in the research. The first way is called "informed consent," where doctors ask the patient and/or family if they want to participate in the research before the surgery. If they participate in the research, they may undergo an experimental operation. This must be done quickly, as people must be operated on within eight hours of the onset of brain hemorrhage. The second way is "deferred consent," where the doctor assesses whether the patient needs surgery if they meet the treatment criteria. Afterwards, the family or patient is asked if the knowledge gained from the surgery can be included in the research.

Deferred consent has so far been seen as positive by all participants. According to participants, it solves some ethical problems that exist with informed consent:

1. Family members must make the decision under great stress in the case of informed consent. Some relatives indicate that they do not feel they have a choice because they cannot fully assess the pros and cons in a short amount of time. Stress and emotions also play a large role, making the decision not well-considered.
2. The time pressure to obtain informed consent is too high. Informed consent delays surgery because the operation is not prepared until after consent is obtained. Potential participants are lost because they cannot be included in time.
3. Due to time pressure and stress, trust in the doctor is a determining factor, giving the doctor a lot of power. Ideally, the patient and family make a choice in an informed and equal manner.
