# Supplementary material for: Participatory methods used in the evaluation of medical devices: a comparison of focus groups, interviews, and a survey
Source: BMC Health Serv Res. 2024 Apr 12;24:462. doi: 10.1186/s12913-024-10887-3 (PMC11015660; doi:10.1186/s12913-024-10887-3)
Supplement: Supplementary file 3 — Supplementary Material 3. [file 12913_2024_10887_MOESM3_ESM.docx]

Supplementary file 3. Coding book interaction
*This file is submitted in accordance with the SAGE author guidelines for supplemental material*

**Code book interaction**

Who?

A1 Moderator > Participants

A2 Participant > Moderator

B Participant > Participant

C1 Moderator - presenting opinions of other participants > Participants

C2 Participants reacting to views of other participants > Moderator

How?

Q&A

1. Question

2. Follow-up question
3. Answer

Topic

4. Introduction of new topic / thematics —shifts the content of the discussion

5. Expansion—shares new aspects of existing topic

6. Differentiation—compares different aspects of topics

Interpersonal Connections

7. Agreement—reinforcing another participant’s statements

8. Disagreement—disputing another participant’s statements

9. Support—sympathizing with another participant’s statements
