# Supplementary material for: Participatory methods used in the evaluation of medical devices: a comparison of focus groups, interviews, and a survey
Source: BMC Health Serv Res. 2024 Apr 12;24:462. doi: 10.1186/s12913-024-10887-3 (PMC11015660; doi:10.1186/s12913-024-10887-3)
Supplement: Supplementary file 4 — Supplementary Material 4. [file 12913_2024_10887_MOESM4_ESM.docx]

Supplementary file 4. Calculation feasibility
*This file is submitted in accordance with the SAGE author guidelines for supplemental material*

**Focus group**

*Preparation, data collection and analysis* **Over the course of 7 months
Number of hours worked: 95**

**Preparation**

**Over the course of 5 months
Number of hours worked: 26**

Preparing interview protocols

*March - May 2020*

- 2 hours drafting protocol, 1 hour discussion with two researchers; 1 hours redrafting protocol, 1 hour discussion with two researchers: **7 hours in total**

Recruitment

*May, June, August, September 2020*

- 1 hour discussion list of participants with two researchers, calling and mailing 18 times (45 minutes per participant): **14 hours in total**

Preparing with moderators

*September 2020*

- 1 hour preparation, 1 hour discussion with one researcher and three moderators: **5 hours in total**

**Data collection
Over the course of 1 evening
Number of hours worked: 15 hours**

Focus groups

*September the 23th, 2020*

- 2 hours by five researchers/moderators: **10 hours in total**

Evaluation

*September the 29th, 2020*

- 2 hours by five researchers/moderators: **5 hours in total**

**Analysis**

**Over the course of 2 months
Number of hours worked: 54**

Transcribing interviews
*September – October 2020*

- 5 transcripts of 45 minutes (transcribing takes on average 5 hours per 1-hour transcript): **19 hours in total**

Coding and constructing themes
*September – October 2020*

- 3 hours per transcript: ***15 hours in total***
- Constructing themes: September and November
- 3 sessions of 4 hours; 2 discussions of 2 hours with 2 researchers ***20 hours in total***

**Interviews**

*Preparation, data collection and analysis*

**Over the course of 15 months
Number of hours worked: 315**

**Preparation**

**Over the course of 5 months
Number of hours worked: 26**

Preparation

*March, April, May 2020*

- Preparing interview protocols: 2 hours drafting protocol, 1 hour discussion with two researchers; 1 hours redrafting protocol, 1 hour discussion with two researchers: **7 hours in total**

Recruitment

Round 1

*January, February, May 2020*

- Discussion invitation list (1 hour, 2 researchers): **2 hours in total**
- Invitations round 1: 30 minutes per participant, 17 participants: **8,5 hours in total**

Round 2
*September, October, November, January 2021-22*

- Invitations round 2: 30 minutes per participant, 17 participants: **8,5 hours in total**

**Data collection
Over the course of 10 months** *Overlap with analysis* **Number of hours worked: 45**

Round 1
*November 2020 to May 2021*

- 1 hour per interview on average. 30 minutes preparation per interview. During two interviews two researchers were present: **28 hours in total**

Round 2
*September 2021 to February 2022*

- 1 hour per interview on average. 30 minutes preparation per interview. 11 interviews: **17 hours in total**

**Analysis**

**Over the course of 10 months** *Overlap with data collection* **Number of hours worked: 244**

Round 1
*November 2020 – May 2021*

Round 2
*September 2021 – January 2022*

Transcribing interviews

*November 2020 – June 2021*

- 28 transcripts in both rounds (transcribing takes on average 5 hours per 1-hour transcript): **140 hours in total**

Coding and constructing themes

*November 2020 – January 2022*

- Coding: 3 hours per transcript: **84 hours in total**
- Constructing themes: 3 sessions of 4 hours; 2 discussions of 2 hours with 2 researchers: **20 hours in total**

**Survey***Preparation, data collection and analysis*

**Over the course of 6 months
Number of hours worked: 81**

**Preparation**

**Over the course of 9 months
Number of hours worked: 57**

Preparation

*March, April, May, September, October, November 2020*

- Drafting and discussing protocol: 12 hours drafting protocol, 2 times discussion of 1 hour with 2 researchers: protocol, 1 hour discussion with two researchers: **16 hours in total**

Editing surveys in Castor
*March, April, May, September, October, November 2020*

- Editing: **12 hours in total**
- 1 hour discussion with two researchers, 1 hour editing; 1 hour discussion with two researchers, 1 hour editing: **6 hours in total**

Recruitment
*September, October, November 2020*

- Inviting 79 persons, 15 minutes per person: **19 hours in total**
- Communication with 50 participants, 5 minutes each: **4 hours in total**

**Data collection
Over the course of 4 months
Number of hours worked: 7**

Sending invitations

*November 2020 to February 2021*

- Sending invitation link to 76 persons, 5 minutes each: **7 hours in total**

**Analysis**

**Over the course of 1 month
Number of hours worked: 17**

Constructing codes and themes

*September 2021*

- Coding: 3 hours per stakeholder group: **9 hours in total**
- Constructing themes: 1 sessions of 4 hours; 2 discussions of 2 hours with 2 researchers: **8 hours in total**
