# Supplementary material for: Participatory methods used in the evaluation of medical devices: a comparison of focus groups, interviews, and a survey
Source: BMC Health Serv Res. 2024 Apr 12;24:462. doi: 10.1186/s12913-024-10887-3 (PMC11015660; doi:10.1186/s12913-024-10887-3)
Supplement: Supplementary file 5 — Supplementary Material 5. [file 12913_2024_10887_MOESM5_ESM.docx]

| **Perspective** | **Type of stakeholder** | Focus group  Invited | **Focus group Enrolled** | Interview  Invited | **Interview Enrolled** | Survey  Invited | **Survey Enrolled** | Total  Invited | **Total Enrolled** |
| --- | --- | --- | --- | --- | --- | --- | --- | --- | --- |
| Patient | Patients | 6 | **4** | 4 | **3** | 15 | **11** | 25 | **18** |
|  | Relatives of patients | 3 | **2** | 7 | **4** | 14 | **10** | 24 | **16** |
| Care | Neurologists | 1 | **1** | 2 | **1** | 7 | **5** | 10 | **7** |
|  | Neurosurgeons | 1 | **1** | 1 | **4** | 5 | **3** | 7 | **8** |
|  | Other relevant specialists (Radiologist, ICU-specialist) | 3 | **2** | 4 | **2** | 9 | **4** | 16 | **8** |
|  | Nurses, other hospital personnel | 2 | **2** | 3 | **1** | 10 | **6** | 15 | **9** |
| Policy | Funding organisation representatives | 2 | **2** | 0 | **0** | 4 | **3** | 6 | **5** |
|  | Policy makers | 3 | **2** | 1 | **1** | 10 | **1** | 14 | **4** |
|  | Industrial partners | 2 | **2** | 0 | **0** | 1 | **0** | 3 | **2** |
|  | Insurance/reimbursement | 1 | **0** | 1 | **1** | 4 | **0** | 6 | **1** |
|  |  | 24 | **18** | 23 | **17** | 79 | **43** | 126 | **78** |

Supplementary file 5. Overview of types and number of invited and enrolled participants in each data-collection method

*This file is submitted in accordance with the SAGE author guidelines for supplemental material*
