# Supplementary material for: Participatory methods used in the evaluation of medical devices: a comparison of focus groups, interviews, and a survey
Source: BMC Health Serv Res. 2024 Apr 12;24:462. doi: 10.1186/s12913-024-10887-3 (PMC11015660; doi:10.1186/s12913-024-10887-3)
Supplement: Supplementary file 6 — Supplementary Material 6. [file 12913_2024_10887_MOESM6_ESM.docx]

Supplementary file 6. Overview of themes in each method
*This file is submitted in accordance with the SAGE author guidelines for supplemental material*

|  | **Themes prevalent in method** | | | | | **Themes addressed by multiple stakeholder groups in method** | | | | |
| --- | --- | --- | --- | --- | --- | --- | --- | --- | --- | --- |
|  | **Interviews round 1** | **Interviews round 2** | **Focus groups round 1** | **Focus groups round 2** | **Survey** | **Interviews round 1** | **Interviews round 2** | **Focus groups round 1** | **Focus group round 2** | **Survey** |
| **Improvements in hospital care** |  |  |  |  |  |  |  |  |  |  |
| **Treatment broadly** |  |  |  |  |  |  |  |  |  |  |
| Problems with hospital room | x |  | x | x | x | x |  |  |  |  |
| Problems with hospital bed |  |  |  |  | x |  |  |  |  |  |
| Localisation patients – e.g., bringing them to correct rooms | x |  |  | x | x |  |  |  |  |  |
| Shortages of medical equipment | x | x |  |  | x |  |  |  |  |  |
| Problems with online patient portal |  |  |  |  | x |  |  |  |  |  |
| Provision information needs to be aligned with needs patients and family | x | x |  |  |  |  |  |  |  |  |
| Diagnosis needs to be acquired faster | x |  | x |  |  |  |  |  |  |  |
| Shortage of physicians with expertise ICH | x | x | x |  | x | x |  |  |  |  |
| Treatment is not optimally evidence-based | x | x |  |  | x |  |  |  |  |  |
| Problems with blood pressure during treatment | x |  | x |  |  | x |  |  |  |  |
| Current treatments are not effective |  |  |  |  | x |  |  |  |  |  |
| Patients need to be stimulated more often |  |  | x | x |  |  |  |  |  |  |
| Different aetiologies of ICH cause problems during treatment |  |  | x |  |  |  |  |  |  |  |
| Current treatment causes damage | x |  |  |  | x |  |  |  |  |  |
| Satisfaction with treatment |  |  |  |  | x |  |  |  |  |  |
| Trust in physicians |  |  |  |  | x |  |  |  |  |  |
| **Patient transfer from hospital to other facilities** |  |  |  |  |  |  |  |  |  |  |
| Transfer went too fast | x | x |  |  |  |  |  |  |  |  |
| Problems during transfer | x |  | x | x | x |  |  |  | x |  |
| **Cooperation between healthcare professionals** |  |  |  |  |  |  |  |  |  |  |
| Problems in cooperation between healthcare institutions | x |  | x | x |  | x |  |  | x |  |
| Problems in cooperation between specialists | x |  | x | x | x | x |  | x |  |  |
| Problems in cooperation between specialists and nursing staff |  |  | x |  | x |  |  |  |  |  |
| **After-care** |  |  |  |  |  |  |  |  |  |  |
| Rehabilitation therapy needs to be introduced in hospital | x | x | x |  | x | x | x | x |  |  |
| **Prevention** |  |  |  |  |  |  |  |  |  |  |
| Public needs to be informed about problems high blood presure |  |  |  | x | x |  |  |  |  |  |
| **Nursing** |  |  |  |  |  |  |  |  |  |  |
| Shortage of nursing staff |  |  | x |  | x |  |  |  |  |  |
| Many different nurses provide care | x |  |  |  | x |  |  |  |  |  |
| Quality of nursing differs between staff | x |  |  |  | x |  |  |  |  |  |
| Trust in nursing staff |  |  |  |  | x |  |  |  |  |  |
| **Policy** |  |  |  |  |  |  |  |  |  |  |
| Cooperation between different institutions |  |  | x |  | x |  |  |  |  |  |
| ‘Advanced care planning’ can lead to better decisions in treatment | x |  |  |  |  | x |  |  |  |  |
| **Improvements in communication** |  |  |  |  |  |  |  |  |  |  |
| **Topics** |  |  |  |  |  |  |  |  |  |  |
| Shared decision making | x | x |  | x | x | x | x |  |  | x |
| Psychosocial aspects of living with ICH | x | x |  |  |  |  |  |  |  |  |
| Perspective on life after hospital care trajectory | x | x |  |  | x | x | x |  |  |  |
| Aftercare | x | x |  |  | x |  | x |  |  |  |
| Transfer from hospital to subsequent facility | x |  |  |  |  |  |  |  |  |  |
| **Communication style** |  |  |  |  |  |  |  |  |  |  |
| Needs to match the knowledge and competencies of patients and relatives | x | x | x | x |  | x | x | x |  |  |
| Less formal and direct and with more empathy | x | x | x | x | x | x | x |  | x |  |
| Communication needs to be offered more actively | x | x | x | x |  |  | x |  |  |  |
| More repetition is needed | x | x | x | x |  |  | x |  | x |  |
| Communication needs to be offered more frequently | x | x |  |  | x |  |  |  |  |  |
| Communication is offered too late |  |  | x | x |  |  |  |  | x |  |
| Communication needs to be offered in phases |  |  | x | x |  |  |  |  | x |  |
| **Who need to be involved** |  |  |  |  |  |  |  |  |  |  |
| Family needs to be involved more often | x | x | x | x |  |  | x |  |  |  |
| With one contact person or care manager | x | x | x | x | x |  | x | x | x |  |
| With treating physician | x | x |  |  |  |  |  |  |  |  |
| **Improvements in research minimally invasive surgery** |  |  |  |  |  |  |  |  |  |  |
| **In- and exclusion criteria for research** |  |  |  |  |  |  |  |  |  |  |
| Deep-seated brain area’s: expected to cause large improvement | x | x |  |  |  |  |  |  |  |  |
| Frontal and lobar bleeds: inclusion | x |  |  |  |  |  |  |  |  |  |
| Ventricular bleed only with drain | x |  |  |  |  |  |  |  |  |  |
| Age | x | x |  |  |  |  |  |  |  |  |
| Volume | x | x |  |  |  |  |  |  |  |  |
| Location depends on volume of haemorrhage | x |  |  |  |  |  |  |  |  |  |
| Contra-indications that need to be excluded | x | x |  |  |  |  | x |  |  |  |
| Loss of linguistic abilities: exclusion | x | x |  |  |  |  |  |  |  |  |
| Loss of vital functions: low and high levels: exclusion | x | x |  |  |  |  |  |  |  |  |
| Arteriovenous malformation: no consensus | x | x |  |  |  |  |  |  |  |  |
| Composition of study population: generic or selective | x | x |  |  |  |  | x |  |  |  |
| Timing | x | x |  |  | x |  | x |  |  |  |
| Choice in- or exclusion needs to be made by physician | x | x |  |  |  |  |  |  |  |  |
| Choice in- or exclusion needs to be made by patients and relatives | x |  |  |  |  |  |  |  |  |  |
| **Outcome measures** |  |  |  |  |  |  |  |  |  |  |
| Survival | x | x | x |  |  |  | x |  |  |  |
| Volume of surgical evacuation | x | x | x |  | x |  |  |  |  | x |
| Safety | x | x | x |  | x | x |  |  |  | x |
| Disability | x | x | x | x | x | x | x |  |  | x |
| Quality of life of patients | x | x | x |  | x | x | x | x |  | x |
| Quality of life of relatives | x | x |  |  |  | x | x |  |  |  |
| Duration of care trajectory | x | x |  |  |  | x | x |  |  |  |
| Cost-effectiveness | x | x | x |  | x | x | x |  |  | x |
| Usability |  |  |  |  | x |  |  |  |  | x |
| Accessibility of the healthcare system | x | x |  |  |  |  |  |  |  |  |
| Ethical considerations |  |  |  |  | x |  |  |  |  | x |
| **Informed consent** |  |  |  |  |  |  |  |  |  |  |
| Informed consent | x | x |  |  | x | x | x |  |  |  |
| Deferred consent | x | x |  |  |  | x | x |  |  |  |
| **Other aspects research** |  |  |  |  |  |  |  |  |  |  |
| Methodological aspects | x | x | x | x | x | x | x |  |  |  |
| Financial aspects | x |  | x |  |  |  |  |  |  |  |
| Implementation of minimally invasive surgery |  |  | x | x | x |  |  |  |  |  |
| Attitude to minimally invasive surgery | x |  |  |  | x |  |  |  |  |  |
| Attitude to research into minimally invasive surgery |  |  |  |  | x |  |  |  |  |  |
| **N** |  |  |  |  |  |  |  |  |  |  |
| 72 | 58 | 40 | 30 | 19 | 42 | 20 | 22 | 5 | 7 | 9 |
| **Percentage** |  |  |  |  |  | 34% | 58% | 17% | 37% | 21  % |
